# Real-time visualization of thrombus formation at the interface between connectors and tubes in medical devices by using optical coherence tomography

**DOI:** 10.1371/journal.pone.0188729

**Published:** 2017-12-07

**Authors:** Yuki Matsuhashi, Kei Sameshima, Yoshiki Yamamoto, Mitsuo Umezu, Kiyotaka Iwasaki

**Affiliations:** 1 Department of Integrative Bioscience and Biomedical Engineering, Graduate School of Advanced Science and Engineering, Waseda University, Shinjuku, Tokyo, Japan; 2 Department of Modern Mechanical Engineering, Graduate School of Creative Science and Engineering, Waseda University, Shinjuku, Tokyo, Japan; 3 Cooperative Major in Advanced Biomedical Sciences, Graduate School of Advanced Science and Engineering, Waseda University, Shinjuku, Tokyo, Japan; Worcester Polytechnic Institute, UNITED STATES

## Abstract

**Background:**

Blood-contacting devices have contributed to improving the treatment of patients. However, thrombus formation at the interface between a connector and tube is still a potential source of thrombus-related complications that induce stroke or myocardial infarction. We aimed to develop a non-blood-contacting real-time method for visualizing thrombus formation, and to experimentally investigate the time-dependent phenomenon of thrombus formation at the interface between a connector and a tube in a medical device.

**Methods and findings:**

An optical coherence tomography device with a center wavelength of 1330 nm was used to visualize thrombus formation during porcine blood circulation for 50 min in a closed 50-mL circulation system isolated from ambient air. The thrombus formation sites at the interface between a tube and connector were visualized. The area of the thrombus formation at the interface between the inlet of the connector and the tube was found to be 0.012 ± 0.011 mm^2^. Conversely, at the interface between the outlet of the connector and the tube, the area was found to be 0.637 ± 0.306 mm^2^. Thus, significantly larger amounts of thrombus were formed at the outlet interface (p < 0.01). The thrombus formation area at the outlet interface increased over time. Conversely, the area of thrombus formation showed repeated increasing and decreasing behavior at the inlet interface. Flow visualization with particle image velocimetry showed the presence of a flow separated area in the minimal flow phase at the inlet interface and a large recirculating slow flow region at the outlet interface in the minimal flow phase. These data suggested that the recirculating stagnant flow region contributed to thrombus growth.

**Conclusions:**

The method presented here was effective in quantitatively assessing time-dependent phenomena of thrombus formation at the connector-tube interface. The method may contribute to the assessment of thrombogenicity of a novel design of connector.

## Introduction

Blood-contacting medical devices, such as extracorporeal membrane oxygenation for long-term support of respiratory as well as cardiac functions and continuous hemofiltration devices for renal failure patients, are commonly used in various patient treatment procedures [1,2]. However, when a thrombus forms in a medical device, it can potentially lead to complications such as stroke or myocardial infarction [3], or require device replacement [4]. The interface between the connector and the tube has been identified as a frequent thrombus formation site [5]. However, a strategy to prevent thrombus formation is yet to be established. We believe that understanding the time-dependent phenomenon of the thrombus formation process may contribute to the development of implantable devices to reduce thrombus-related complications.

Intravascular ultrasound, angioscopy, intravascular optical coherence tomography (OCT), and magnetic resonance imaging have all been used to observe thrombi in clinical practices [6]. The spatial resolution of intravascular ultrasound ranges from 0.1 to 0.2 mm, whereas that of magnetic resonance imaging is approximately 0.8 mm. Thus, neither of these techniques is sensitive enough to detect the initiation of thrombus formation [6]. In comparison, the spatial resolutions of angioscopy and intravascular OCT are higher, ranging from 0.01 to 0.05 mm. However, both these methods require the removal of some blood components and filling with saline in the vicinity of the observation region, which therefore limit their applicability for continuous monitoring of the thrombus formation process [6].

Previous studies have determined that the optical properties of blood are influenced by the various components of blood cells and plasma, flow conditions, and blood aggregation [7]. Additionally, it is known that the intensity of light scattered from illuminated blood varies instantaneously, especially owing to the changes in the shape of blood components due to shear stress caused by circulation [8]. The magnitude of light scattering from erythrocytes is predominant by two or three orders in comparison with other blood components because of the larger volume and size of the red blood cells [9]. At the thrombus formation site, erythrocytes are trapped because of the presence of adhered platelets and/or a fibrin network [10]. The time-dependent variation of the scattered light intensity from erythrocytes is restricted at the thrombus formation site compared with that in flow circulation. Therefore, the areas with no time-dependent variation in light scattering from blood components can be regarded as thrombus formed areas. It is known that a wavelength between 400 and 600 nm is strongly absorbed by hemoglobin in red blood cells [11]. Deeper imaging in whole blood including scattering blood-cell components can be achieved by employing a longer wavelength, because the absorption with blood cell components decreases with increasing wavelength. However, a longer wavelength is restricted by an increased optical absorption by water in blood [12]. Some researchers have reported the optical imaging of red blood cells using OCT with a center wavelength of 1330 nm [13] [14].

In this study, we aimed to develop a non-invasive, non-blood-contacting, and real-time thrombus-formation visualization method of using non-diluted whole blood. An OCT device with a center wavelength of 1330 nm was used to reduce the attenuation of light intensity by the hemoglobin in erythrocytes [7]. Furthermore, we aimed to experimentally investigate the time-dependent phenomenon of thrombus formation at the interface between a connector and a tube in a medical device.

## Materials and methods

### Real-time thrombus visualization system by using OCT

**Fig 1:**
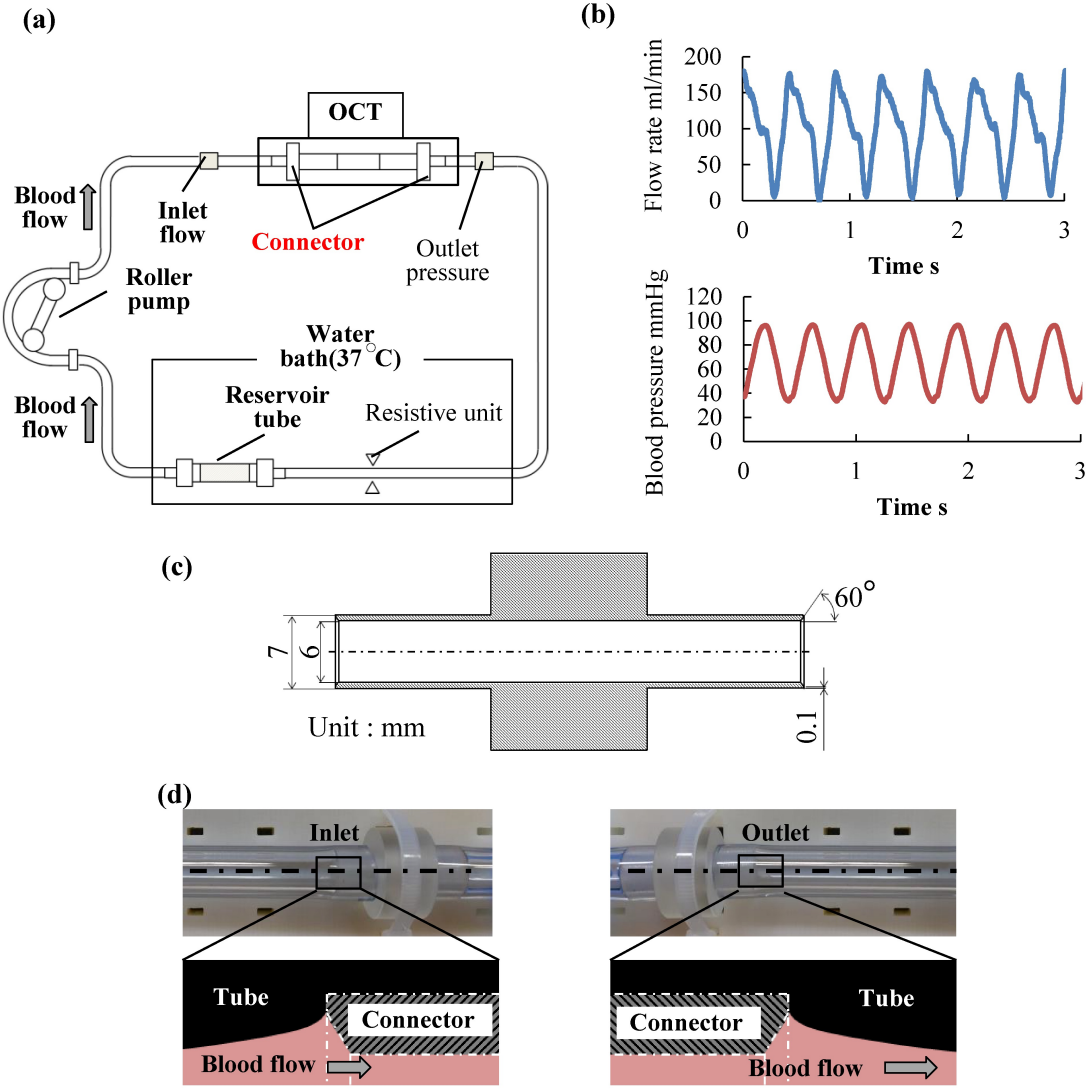
Schematic of a sequential visualization system for thrombus formation by using optical coherence tomography (OCT). (a) Non-blood-contacting sequential visualization system of thrombus formation in a blood circulation system with OCT. (b) Flow and pressure waveforms in the blood circuit. (c) Connector used in this study. (d) Cross-sectional views at the interface between the connector and tube.

We have developed a system that uses OCT for the real-time non-blood-contacting visualization of thrombus formation at the interface between a connector and a tube (Fig 1). A swept-source OCT (Panasonic Healthcare, Tokyo, Japan) with a center wavelength of 1330 nm and a maximum energy of 15 mW was employed to achieve a deeper imaging range and faster scanning speed than ultrasound, angioscopy, and conventional spectral-domain OCT systems.

Image acquisition was performed over a 20 × 20 mm area at the interfaces between the connector and tube. The spatial and time resolutions of the OCT are 0.01 mm and 7 frames per second, respectively. Connectors with inner and outer diameters of 6 and 7 mm, respectively, with 60° taper and tip width of 100 μm were fabricated (Fig 1c). The connectors were made of polyurethane (SG7101 AT, SG7101 B, SID Co. Ltd., Saitama, Japan). A clinical-grade polyvinylchloride (PVC) tube with an inner diameter of 6 mm (Tygon^®^, ACFJ00007, Saint-Gobain S.A., Paris, France) was used. The luminal surfaces of the PVC tubes and connectors were coated twice with segmented polyurethane (Miractran^®^, Nihon Unipolymer, Tokyo, Japan) and then coated once with 2-methacryloyloxyethyl phosphorylcholine (MPC) [15]. The thickness of the coatings was approximately 3 μm. MPC is a polymer that is inert to protein adsorption of blood plasma and platelet adhesion [16,17], and is recognized as a blood-compatible material [18]. MPC is used for coating on the blood-contacting surface of a ventricular assist device, and its excellent anti-thrombogenicity has been shown in clinical practice [19, 20]. In this study, the MPC coating was applied on the luminal surface of the circuit to achieve anti-thrombogenicity of the circuit. A compliant reservoir tube with a diameter and length of 12 mm and 30 mm, respectively, was fabricated by using a dipping method using a blood-compatible segmented polyurethane (TM-5; Nipro Co. Ltd., Osaka, Japan). For the compliant tube, MPC was coated once. The coating procedure and fabrication of the compliant tube were conducted in a class 100 clean room. The diameters of the connectors and tubes were matched with those of the circuits for continuous hemofiltration therapies.

The connector and tube were joined and placed in a closed 50-mL circulation system isolated from ambient air. Typical cross-sectional images at the inlet and outlet interfaces between the connector and the tube are shown in Fig 1d. This system consisted of a roller pump (Masterflex 07528; Yamato Scientific Co. Ltd., Tokyo, Japan), the compliant reservoir tube, PVC tubes, and a resistive unit (Fig 1a). All components of the system were sterilized with ethylene oxide gas before the tests. Flow and pressure were monitored by using an electromagnetic flow meter (FF-060T; Nihon Kohden, Tokyo, Japan) and a pressure transducer (UK-801; Baxter, Irvine, CA, USA). In this study, the mean flow rate and pressure were adjusted to 100 mL/min and 70 ± 2 mm Hg, respectively (Fig 1b), considering the clinically-relevant conditions in continuous hemofiltration therapy in Japan. The reservoir tube and a part of the PVC tube were placed in a water bath set to 37°C to simulate the temperature of circulating blood (Fig 1a).

### Blood sampling

This study was approved by the ethical committee of Waseda University (2015-A095). Porcine blood was used in this study. Blood was obtained under general anesthesia by administration of isoflurane as an inhalation anesthetic. An intravascular 8-Fr catheter introducer (Radifocus^®^ Introducer H; Terumo Co., Tokyo, Japan) was inserted into a porcine carotid artery. Before drawing blood, 5.2 IU/mL of heparin (Novo-Heparin; Mochida Pharmaceutical Co., Tokyo, Japan) was administered to achieve a clinically-relevant activated clotting time of approximately 200 s [21]. Blood was then drawn into a blood bag (Terumo) by using the gravity drainage method. Blood with a low activated clotting time of approximately 200 s is likely to induce thrombus formation. Therefore, the luminal surface of the blood bag was also pre-coated with MPC to suppress protein adsorption and platelet adhesion.

### Visualization of the thrombus formation process

Blood circulation tests were performed six times using porcine blood collected from six different individuals. The thrombus formation visualization tests were started within 4 h of drawing blood. Porcine whole blood was poured into the circulation system and circulated for 50 min to investigate the initial process of thrombus formation. The direction of the OCT images was perpendicular to the interface between the connectors and tubes. OCT images were taken for 6 s at 10-min intervals. The concentration of platelets and hematocrit in the blood samples was measured with an automated hematology analyzer (Celltac α, Nihon Kohden). After each experiment, the interface between the connectors and tubes was rinsed with phosphate-buffered saline.

### Thrombus visualization method by using OCT images

The average of six consecutive time-difference images taken in 1 s immediately after the start of blood circulation was used to differentiate the still connectors and tubes from those during circulation. Each average image of six consecutive time-difference images was analyzed to eliminate the influence of lighting at the start of the circulation and at 10, 20, 30, 40, and 50 min. Otsu’s method [22] was used to calculate optimum threshold values for differentiating the still area from the area during circulation at each sampling time point. The areas of the connector and tube were subtracted from the extracted images to define the area of thrombus formation.

### Validation of the thrombus visualization method

The thrombus formation area was investigated by comparing the extracted areas using OCT between with whole blood and saline solution. To investigate the observational accuracy in the depth direction, i.e., into the direction away from the luminal surface of the tube, OCT intensities were compared at seven equidistantly spaced regions of the interest (ROIs) from the lumen of the tube up to 840 μm in the depth direction. Each ROI was a 5-pixel square. The pixel size of the OCT images was 20 μm × 20 μm. At each depth away from the tube, the average values of 20 ROIs were calculated. The influence of the depth away from the tube on signal intensities was investigated to understand the light attenuation property.

### Flow visualization at the interface between a connector and a tube by using particle image velocimetry

To investigate the influence of hemodynamics on thrombus formation and thrombus detachment at the interface between a connector and a tube, we performed fluorescent particle image velocimetry (PIV). The PIV system was constructed based on the equivalent circulation system previously described. As an alternative to the PVC tubes at the interface, a silicone model, which comprised a straight flow channel with a diameter of 6 mm, was used to match the refractive index between a working fluid and the silicone model to 1.4096. The refractive index was measured with a refractometer (DR-A1; Atago Co. Ltd., Tokyo, Japan). A mixed solution of glycerin and water (43 wt.%), a Newtonian fluid, with a viscosity of 3.7 mPa ⋅ s was used as the working fluid. The Young’s modulus of the silicone model was set to 2.13 ± 0.08 MPa at room temperature to prevent the expansion of the inner diameter of the flow channel due to the inner circuit pressure. The circulation system was placed inside an acrylic box. Fluorescent particles (Fluostar; EBM, Tokyo, Japan) with the mean diameter and density of 15 μm and 1.1 g/cm^3^, respectively, were used. The acrylic box was filled with the same glycerin solution as the working fluid, but without the fluorescent particles. A high-speed camera (VC13-0192, Imager Pro XL camera; LaVision, Goettingen, Germany) and Nd:YLF laser (DS20-527; Photonics Industries, Bohemia, NY, USA) were used. The PIV images were acquired by using a high-speed camera with a pixel size of 0.025 mm and a frame rate of 400 frames/s. An optical filter (CVI Melles Griot, Albuquerque, NM, USA) with a cutoff frequency of 550 nm was used to reduce the scattered light noise from the rigid silicone model. The effective velocity and spatial resolution of the PIV system were 0.01 mm/s and 0.1 mm × 0.1 mm, respectively.

### Statistical analysis

Statistical analyses were performed by using SPSS (version 21; IBM, Tokyo, Japan). The change in the number of platelets and hematocrit before and after testing was compared by using Student’s t-test. The average differences in the thrombus-formed areas between the connector inlet and connector outlet after circulation were compared by using Student’s t-test. The thrombus formation areas at the interfaces between the connector and tube under whole blood and saline solution were compared by using Student’s t-test.

## Results

### Blood conditions

The activated clotting time at the beginning of the test was 214 ± 35 s. The average hematocrit values before and after the tests were 32.4 ± 1.7% and 31.0 ± 1.6%, respectively. The difference between these values was not significant (p = 0.10). The number of platelets before and after the tests was 32.5 ± 4.1 × 10^4^/μL and 26.6 ± 5.2 × 10^4^/μL, respectively. The platelet concentration was significantly reduced after the test (p < 0.05).

### Observation of the interface between the connector and the tube after replacement of whole blood with phosphate-buffered saline

**Fig 2:**
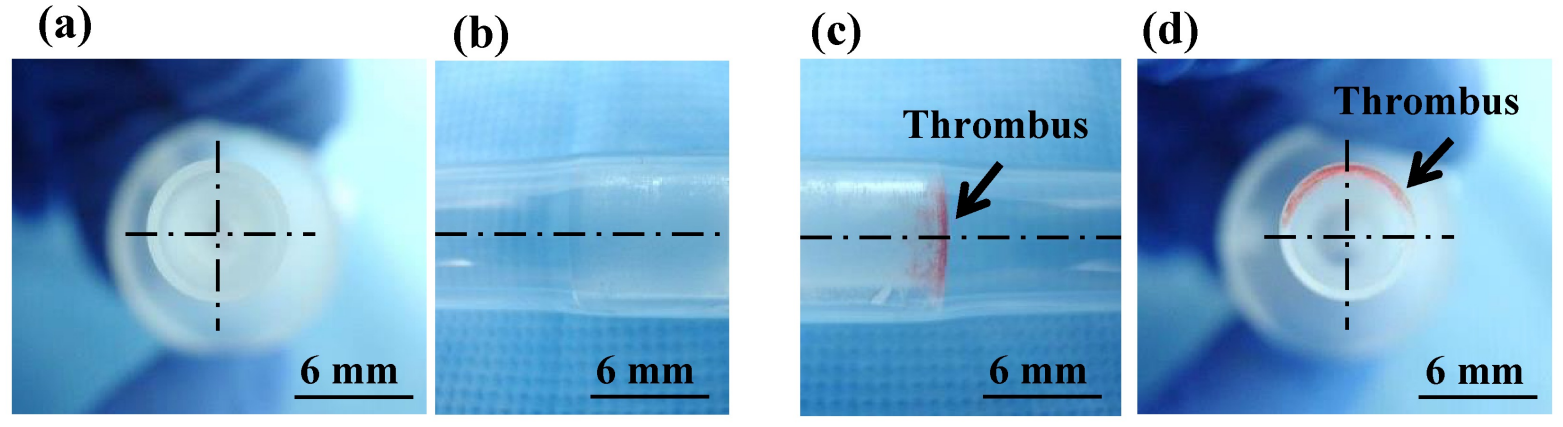
Thrombus formation at the connector interface. (a) Cross-sectional view of the connector inlet. (b) Side view of the connector inlet. (c) Side view of the connector outlet. (d) Cross-sectional view of the connector outlet.

The interface between the connector and the tube during blood circulation for 50 min was observed after a careful rinse with phosphate-buffered saline (Fig 2). In all the six tests, no visible thrombus was observed at the interface between the inlet of the connector and the tube. On the other hand, thrombi consistently formed at the interface between the outlet of the connector and the tube.

### Extraction of thrombi images from OCT images

**Fig 3:**
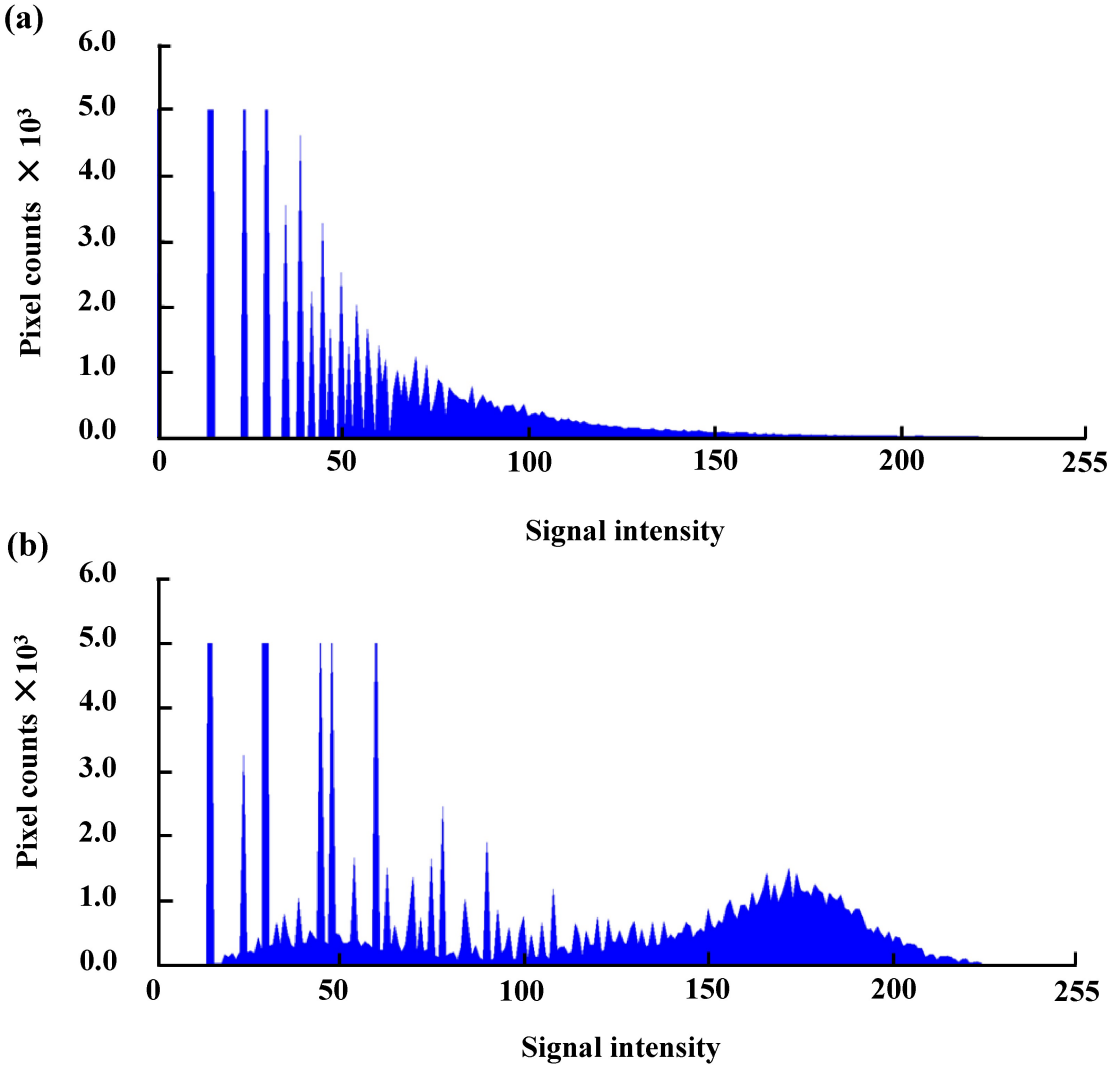
Signal intensity histograms. (a) Signal intensity histogram of the original optical coherence tomography image. (b) Signal intensity histogram of the average of six time-differential images of seven consecutive frames.

**Fig 4:**
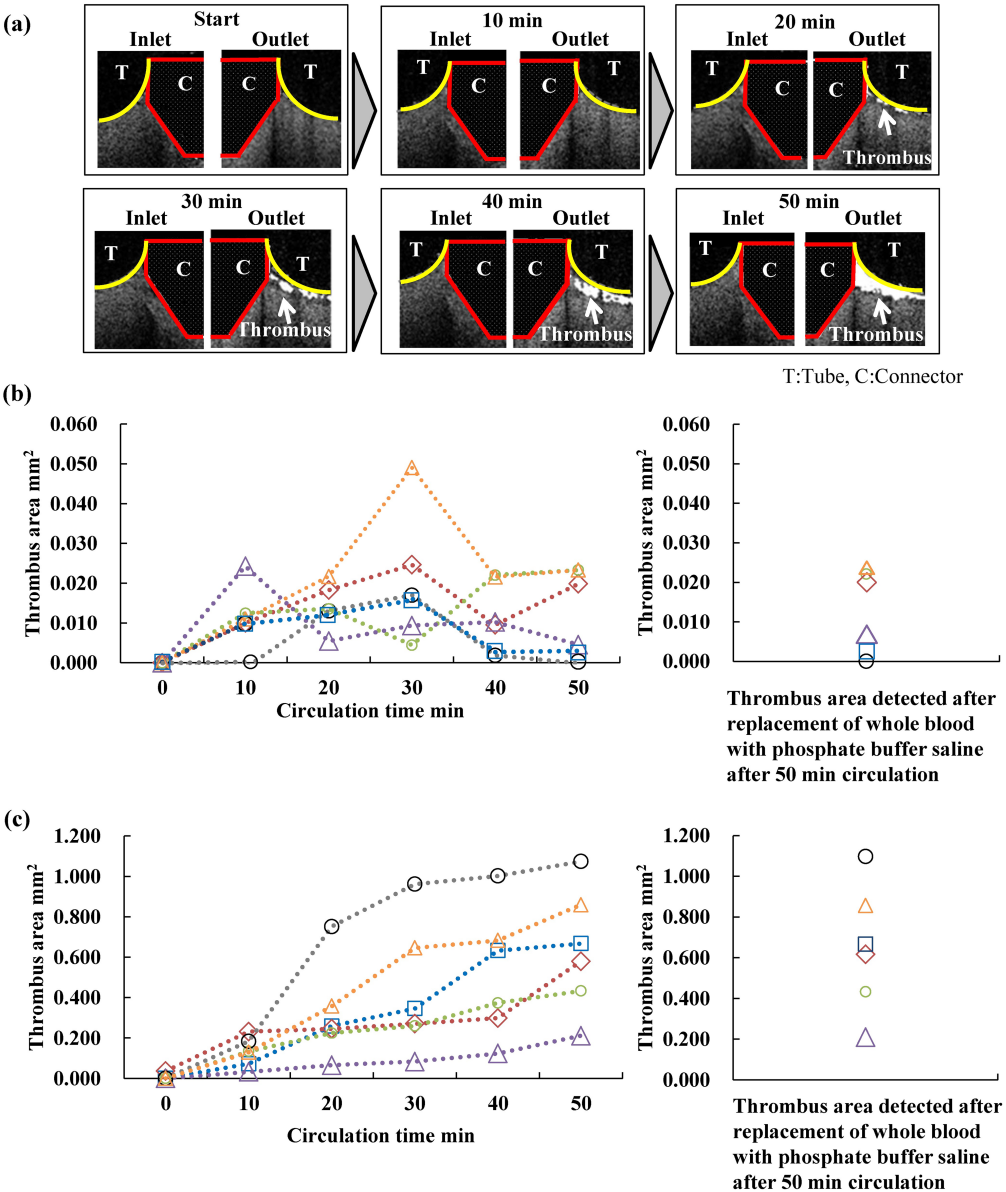
Quantitative changes in the sequential thrombus formation area measured with optical coherence tomography. (a) Thrombus formation at the interface between the connectors and the tube in the blood circulation test. The white arrow indicates the thrombus area, red lines represent the connectors, and yellow lines represent the location of the tube. The data represent one of the six tests shown with blue square plots in (b) and (c). (b) Thrombus formation area at the interface between the inlet of the connector and the tube. The tests were performed six times using porcine blood collected from six different individuals. (c) Thrombus formation area at the interface between the outlet of the connector and the tube. The right sides of (b) and (c) show the areas of thrombus formation detected by OCT after replacing blood with saline solution.

By using the signal intensity histogram of the original OCT images (Fig 3a), the signal intensity histograms of the average of the six time-differential images of seven consecutive frames were obtained (Fig 3b). Accordingly, the sites of the forming thrombi and the blood could be distinguished by calculating the time variation in the signal intensity. Based on the above procedures, the areas of thrombus formation during 10–50 min at 10-min intervals were visualized (Fig 4a).

The sequential changes in the areas of thrombus formation at the interface between the inlet and outlet of the connector and the tube were quantified by using OCT (Fig 4b and 4c). The area of the thrombus formation at the interface between the inlet of the connector and the tube was found to be 0.012 ± 0.011 mm^2^ in a 50-min blood circulation. Conversely, at the interface between the outlet of the connector and the tube, the area of thrombus formation was found to be 0.637 ± 0.306 mm^2^. Thus, there was a significant difference in the area between the inlet and outlet interfaces (p < 0.01). It should also be noted that the thrombus formation area at the outlet interface increased over time. Conversely, the area of thrombus formation showed repeated increasing and decreasing behavior at the inlet interface, although the areas were relatively small compared to those at the outlet interface. The Pearson correlation coefficient between the concentration of the platelets before testing and the total thrombus formation area at the interface between the inlet of the connector and the tube was −0.41. Conversely, the Pearson correlation coefficient between the concentration of platelets before testing and the total thrombus formation area at the interface between the outlet of the connector and the tube was 0.93, which signifies a strong correlation.

### Validation of thrombus visualization

#### Influence of the blood-circulating environment on the detected size of the thrombus formation area

Analysis of the OCT data showed the presence of the areas assumed to be thrombus over time. After the circulation, thrombi were visible at the outlet of the connector and the tube, while no thrombi were visible at the inlet of the connector and the tube. The OCT analysis showed that thrombus formation areas at the interfaces between the connector and tube under whole blood and saline solution were 0.012 ± 0.011 mm^2^ and 0.009 ± 0.008 mm^2^ (p = 0.62) at the inlet interface and 0.637 ± 0.306 and 0.646 ± 0.311 mm^2^ (p = 0.96) at the outlet interface, respectively. Thus, there was no significant difference between the whole blood and saline conditions.

#### Attenuation of signal intensity

**Fig 5:**
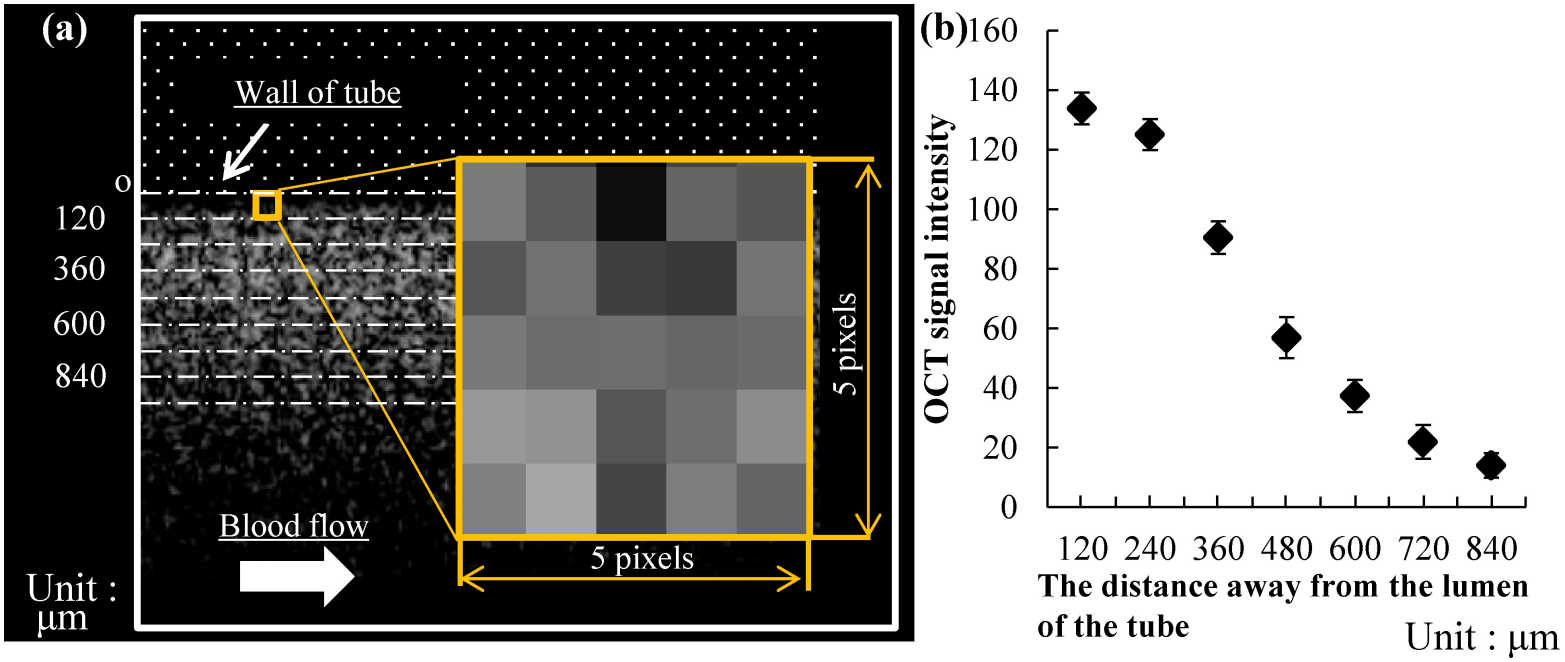
Light attenuation property of the optical coherence tomography due to the depth. (a) The observation region was set from the lumen of the tube toward the depth direction up to 840 μm by each 120-μm interval. (b) Relationship between OCT signal intensities and depth away from the lumen of the tube.

Fig 5 shows the measurement results of the OCT signal intensities at seven equidistant segments located between 120 and 840 μm from the lumen of the tube. The signal intensities (arbitrary units) of the seven ROIs were 130, 125, 90, 56, 37, 22, and 14, respectively. Thus, the signal intensity decreased by 4% at a depth of 240 μm and by 40% at a depth of 480 μm.

#### Flow visualization at the interface between a connector and a tube by using PIV

**Fig 6:**
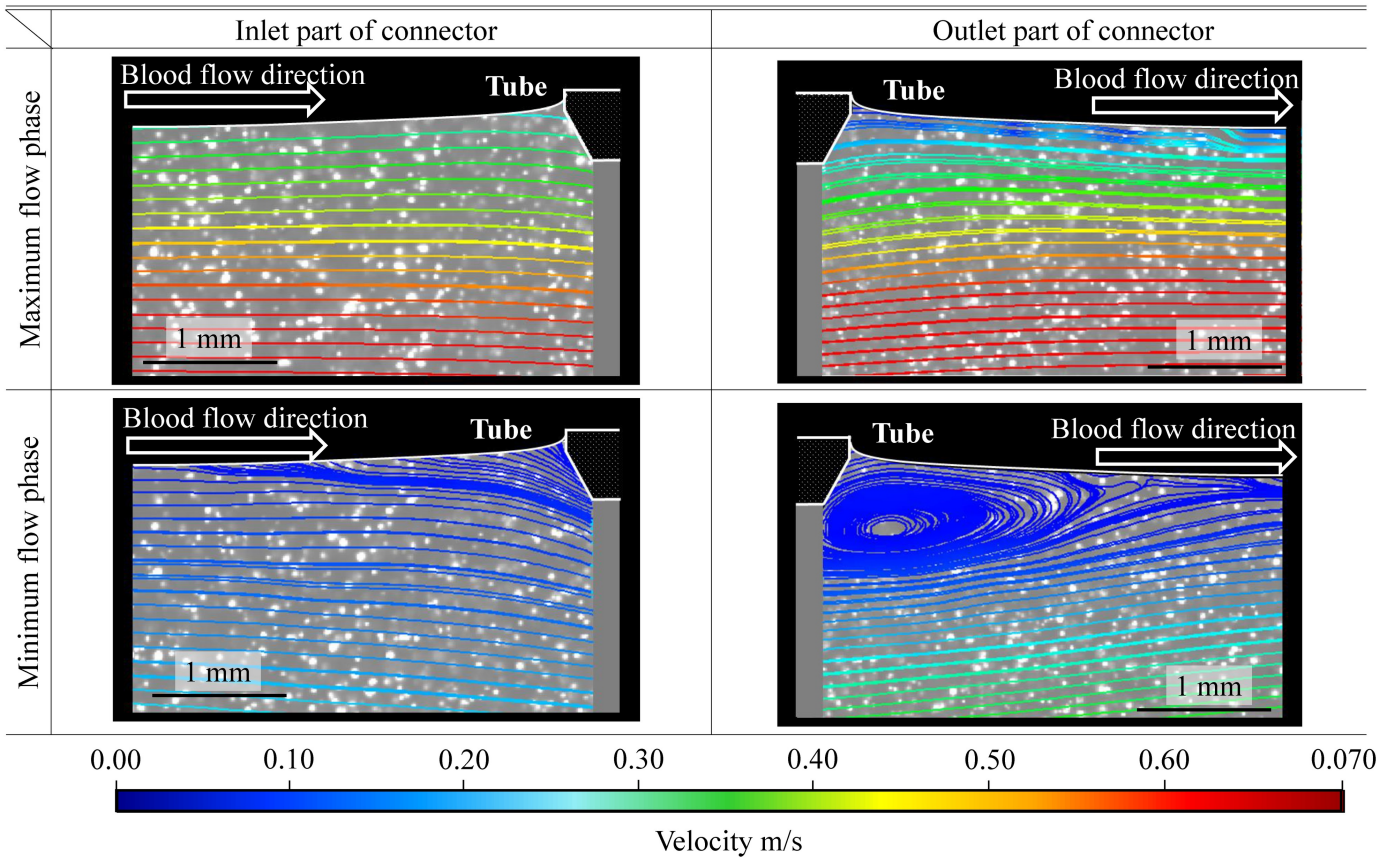
Flow in the vicinity of the interface between the connector and tube. Flow was visualized using particle image velocimetry. The white lines indicate the lumen of the tube and the edge of the connector.

Flow velocity distributions at the interface between the connector and tube were investigated during the pulsatile flow. Flow distribution and streamline at the maximal and minimal flow phases were shown at the interface between the connector and tube, respectively (Fig 6).

A large recirculating stagnant flow region was observed at the interface between the outlet of the connector and tube in the minimal flow phase. At the interface between the inlet of the connector and tube, a flow-separated area was observed in the minimal flow phase. These data suggested that the recirculating stagnant flow region contributed to the growth of the thrombus.

## Discussion

### Development of a method that uses OCT to visualize thrombus formation at the interface between connectors and tubes in real time

In this study, we succeeded in developing a method for visualizing thrombus formation in real time at the interface between a connector and a tube, in whole porcine blood pulsatile flow and pressure conditions. At the interface between the connector inlet and tube, areas of the thrombus formation showed repeated increasing and decreasing behavior, although the areas were relatively small compared to those at the outlet interface. These data suggested that the small thrombus formed at the interface between the connector inlet and tube might be fragile. In clinical situations, the thrombus detached from the interface creates the potential risk for adverse events. From the PIV study, a flow separated area was observed in the minimal flow phase during the pulsatile circulation at the inlet interface. Conversely, the thrombus formation area at the outlet interface increased over time. From the PIV study, a large recirculating slow flow region was observed at the outlet interface in the minimal flow phase. These data suggested that the recirculating stagnant flow region contributed to the growth of the thrombus, while the generation of a slow-velocity flow-separated area during pulsatile circulation became a potential risk of thrombus formation in the minimal flow phase and detachment in the maximal flow phase.

Our real-time thrombus formation visualization method by using OCT under whole blood circulation conditions enabled the quantification of thrombus formation and growth at the interface between the connector and the tube. Medical devices that have tubing connections, such as ventricular assist devices, extracorporeal membrane oxygenation devices, and hemofilters, still have the drawback of increasing the risk of thrombosis [23–26]. In previous studies, thrombus formation could only be observed after a blood treatment or extraction procedure. The method we are developing can investigate the sequential thrombus formation process and detachment phenomena. The findings from the OCT study may contribute to the development of an ideal connector to reduce thrombus formation as well as thrombus detachment.

### Limitations

In this study, red thrombi containing erythrocytes were visualized by analyzing OCT data. However, the ability to visualize white (predominantly fibrin) thrombi was not shown. This could potentially be achieved by using a platelet-rich suspension with plasma by excluding erythrocytes. The OCT imaging of the interface at shorter intervals and in a longer time course may improve the understanding of phenomena of thrombus formation, growth, and detachment. In addition, histological examination of the thrombi formed at the interfaces may improve understanding of time-dependent thrombus-related phenomena. Furthermore, the amount of thrombus detached will be quantitatively investigated by measuring the amount of thrombus present in the circuit after the circulation tests. The developed method successfully captured two-dimensional images of thrombi. The next step is to capture three-dimensional images to determine the exact location of thrombus formation. Achieving this will provide a better understanding of the relationship between hemodynamics and thrombus formation. Three-dimensional images may be captured by developing a stereo-OCT system or by viewing from different directions using the single OCT system.

## Conclusion

We developed a non-blood-contacting real-time visualization method of thrombus formation under whole blood flow conditions by using OCT with a center wavelength of 1330 nm, and experimentally investigated the time-dependent phenomenon related to thrombus formation at the interface between a connector and a tube. The method may contribute to the assessment of thrombogenicity of various connectors and the development of a novel design of connectors to reduce the formation at the interface between connectors and tubes in medical devices.
